# Room Temperature Ionic Liquids as Green Solvent Alternatives in the Metathesis of Oleochemical Feedstocks

**DOI:** 10.3390/molecules21020184

**Published:** 2016-02-06

**Authors:** Priya A. Thomas, Bassy B. Marvey

**Affiliations:** Department of Chemistry, Sefako Makgatho Health Sciences University, P. O. Box 138, Medunsa 0204, South Africa; priyaaregi@gmail.com

**Keywords:** RTILs, green solvents, olefin metathesis, oleochemical feedstocks

## Abstract

One of the most important areas of green chemistry is the application of environmentally friendly solvents in catalysis and synthesis. Conventional organic solvents pose a threat to the environment due to the volatility, highly flammability, toxicity and carcinogenic properties they exhibit. The recently emerged room temperature ionic liquids (RTILs) are promising green solvent alternatives to the volatile organic solvents due to their ease of reuse, non-volatility, thermal stability and ability to dissolve a variety of organic and organometallic compounds. This review explores the use of RTILs as green solvent media in olefin metathesis for applications in the oleochemical industry.

## 1. Introduction

Olefin metathesis was discovered by Banks and Bailey in 1964, while looking for an effective heterogeneous catalyst to replace hydrogen fluoride for converting olefins into high-octane gasoline via olefin-isoparaffin alkylation [1]. When heated with molybdenum in the form of its metal oxide or [Mo(CO)_6_], propylene can be catalytically converted to ethylene and 2-butene. This process was subsequently commercialized as the Philips Triolefin Process. In 1967, Calderon named the reaction metathesis, while converting 2-pentene into 2-butene and 3-hexene using the catalyst system WCl_6_/EtAlCl_2_/EtOH [2]. Metathesis reactions are under thermodynamic control, however, metathesis of a terminal alkene or alkyne displaces the reaction towards the product. The reaction under reduced pressure helps to eliminate volatile olefin and displaces metathesis reaction. Alkene metathesis also leads to the formation of both *Z* and *E* isomers. Many metathesis reactions are also under kinetic control.

**Scheme 1:**
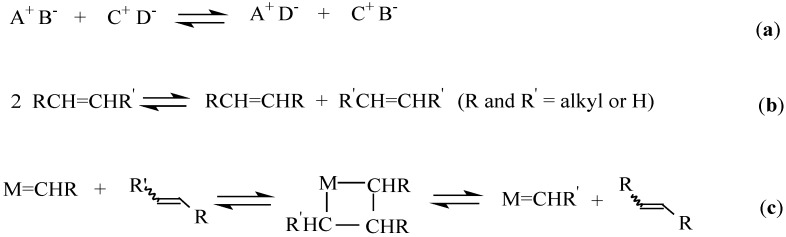
Olefin metathesis reaction (**a**) Ion exchange; (**b**) Olefin metathesis; (**c**) Chauvin mechanism.

Recent advances in olefin metathesis has provided new opportunities for converting oleochemical feedstocks into a variety of high value intermediates which find applications in the polymer, petrochemical, oleochemical and pharmaceutical industries [3,4,5,6]. Olefin metathesis is an alkylidene exchange reaction (Scheme 1) between two reacting alkenes, mediated by transition metal alkylidene complexes [7]. The word metathesis is derived from the Greek word µεταθεσιζ that meaning transposition. The reaction involves the exchange of ions to produce the most stable ion pairs (Scheme 1a) [8]. It follows the Chauvin mechanism in which carbon-carbon double bonds are ruptured and new bonds are formed (**1b**) [9]. The process involves the reaction between an olefin and a transition metal alkylidene complex in a [2+2] fashion to generate an unstable metallacyclobutane intermediate. This cyclobutane intermediate then rearranges to form a new metal carbene and an olefin (Scheme 1b) [9].

**Scheme 2:**
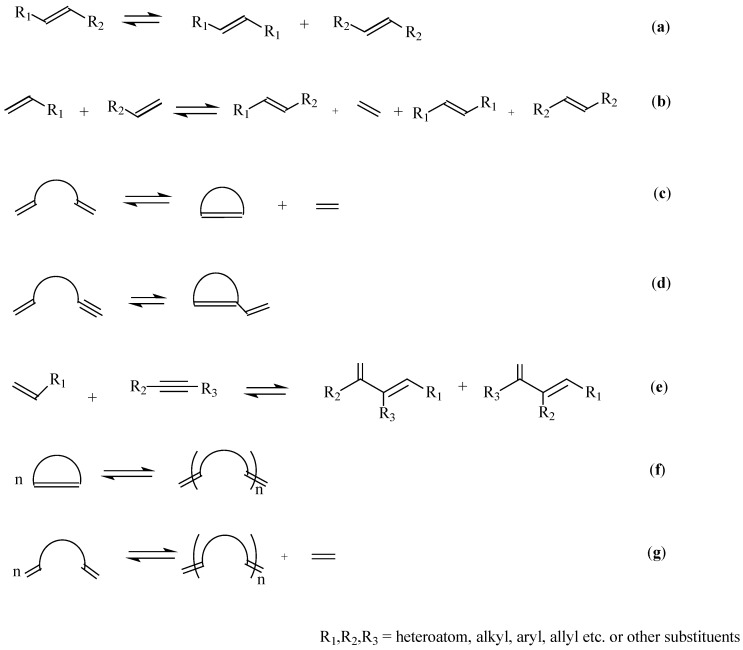
Types of olefin metathesis reaction (**a**) Self-metathesis; (**b**) cross-metathesis; (**c**) Ring-Closing Metathesis (RCM); (**d**) Ring-Closing Ene-Yne Metathesis (RCEYM); (**e**) Ene-Yne Cross-Metathesis (EYCM); (**f**) Ring-Opening Metathesis Polymerisation (ROMP); (**g**) Acyclic Diene Metathesis Polymerisation (ADMET).

Several types of olefin metathesis are identified [10,11] and include self-metathesis (SM), cross-metathesis (CM), ring-closing metathesis, (RCM), ring-opening metathesis (ROM), ring-opening metathesis polymerisation (ROMP) and acyclic diene metathesis polymerisation (ADMET) (Scheme 2). The metathesis reactions that are most widely applied in oleochemistry are SM, CM and ADMET. Using these metathesis reactions, the double bonds present in vegetable oils can be altered leading to the introduction of new functional groups.

## 2. Oleochemical Feedstocks in Olefin Metathesis

**Figure 1:**
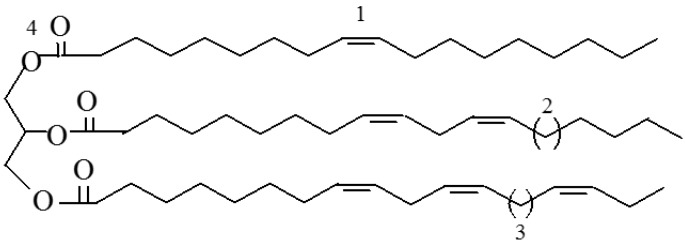
Structure of a triglyceride showing the reactive sites: 1 = double bond; 2 = monoallylic position; 3 = bisallylic position; 4 = ester group.

**Figure 2:**
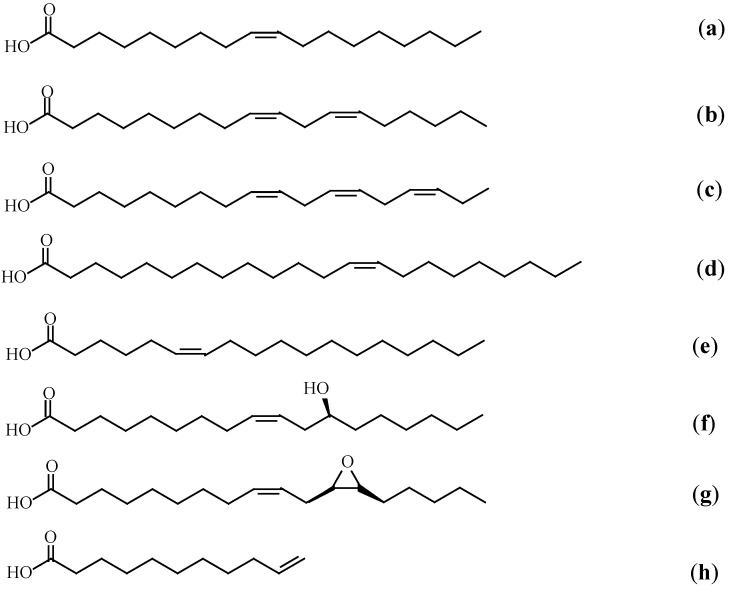
Commonly used fatty acids for the synthesis of renewable platform chemicals (**a**) oleic acid (**b**) linoleic acid (**c**) linolenic acid (**d**) erucic acid (**e**) petroselinic acid (**f**) ricinoleic acid (**g**) vernolic acid (**h**) 10-undecenoic acid.

Fats and oils of animal origin, e.g., fish oils, and oils of vegetable origin, e.g., soybean, linseed, palm and sunflower oils, as well as their derivatives, have been the most important renewable feedstocks that have received a growing attention from the academy and industry in recent years. Vegetable fats contain more unsaturation, allowing for functionalization, and hence are considered to be more healthy than animal fats. The abundance and relatively low cost of these food components make them an interesting raw material for the chemical industry. Vegetable oils are one of the most important sources of polymer precursors. Vegetable oils are composed of acylglycerols or triglycerides (Figure 1) [12], which are the esters of glycerol with three fatty acids. The double bonds, ester groups and allylic positions present in the triglycerides are highly reactive sites, from which a variety of polymers with different structures and functionalities can be prepared. The commonly used fatty acids for the synthesis of renewable platform chemicals are shown in Figure 2 [13,14].

## 3. Overview of Catalyst Systems

**Figure 3:**
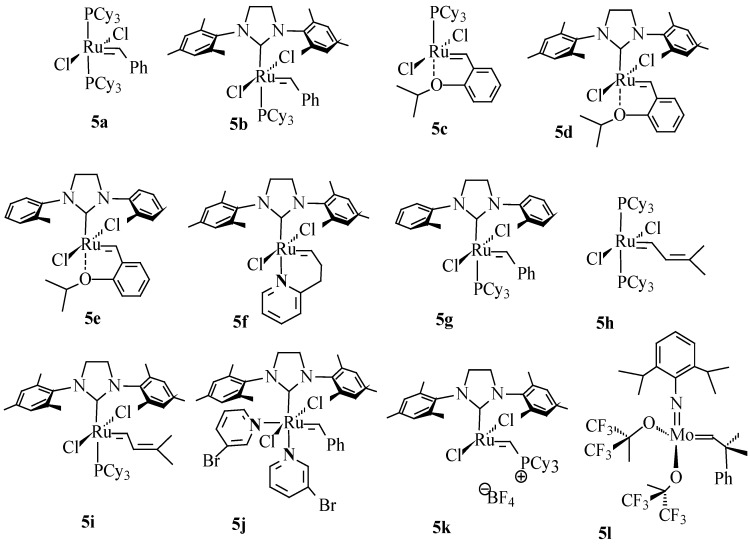
Some commercially available Ru and Mo based metathesis catalysts (Cy = cyclohexyl).

**Scheme 3:**
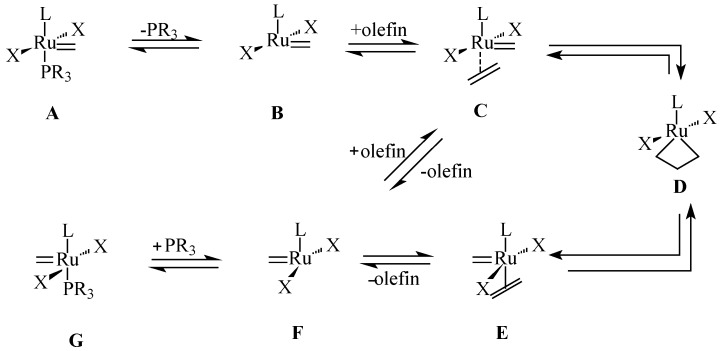
Dissociative mechanism for Ru-catalysed olefin metathesis showing Ruthenium alkylidene (**A,G**); 14e intermediate (**B,F**); 16e species (**C,E**); ruthenacyclobutane (**D**).

Many catalysts have been used for the metathesis reactions. The most widely used are metal alkylidene complex of a transition metal such as molybdenum or ruthenium (Figure 3). Amongst them are Grubbs first generation (**5a**), Grubbs second generation (**5b**), Hoveyda-Grubbs first generation (**5c**), Hoveyda-Grubbs second generation (**5d**), Grubbs and Hoveyda type ruthenium alkylidene (**5e**–**j**); Piers second generation (**5k**) and Schrock molybdenum alkylidene catalysts (**5l**). The complexes of other metals such as tungsten, rhenium and osmium are also used in olefin metathesis, however, these exhibit lower reactivity [15]. Schrock Mo catalyst is generally more reactive than the Ru catalysts and is prepared and handled under inert atmosphere. Ru complexes are more popular than Mo complexes due to their inertness to air and water. Sanford *et al*. [16] have reported the mechanism of ruthenium-catalyzed olefin metathesis reactions (Scheme 3) [17,18]. In the first step, an 14e intermediate (B) is generated from ruthenium alkylidene (A) by the loss of one of the ligands. The metal carbene species (C) reacts with olefin to form an unstable ruthenaclobutane intermediate (D). Finally, the new olefin is formed by the shift in a direction perpendicular to the initial olefin shift.

Grubbs first generation catalyst **5a** containing bulky trialkylphosphine ligands have low thermal stability at 50 °C and low reactivity towards internal olefins. Grubbs second generation catalyst **5b** bearing an *N*-heterocylic carbene ligand such as 1,3-bis(2,4,6-trimethyphenyl)imidazol-2-ylidene (IMes) is thermally more stable than catalyst **5a** and also exhibit higher activity for functionalized and internal olefins [19,20]. The new catalyst bearing a 1,3-dimesityl-4,5-dihydroimidazol-2-ylidene and styrenyl ether ligand, was synthesized based on the accelerating effect of a variety of saturated and unsaturated imidazolin-2-ylidene carbine ligands on the activity of Ru based metathesis catalysts [21].

## 4. RTILs as Green Solvent Alternatives

The demand for environmentally friendly solvents has led to the development of alternative solvents and technologies. In the previous years, water has emerged as a useful reaction medium and has been successfully used in metal catalyzed reactions [22,23,24,25]. However, the low miscibility of organic substrates in water has limited its usage in organic reactions. The usage of perfluorinated solvents has been reported for many organic and catalytic reactions [26,27]. The need for specific ligands to solubilize catalyst in the perflourinated phase and the decomposition of these solvents at high temperature to toxic compounds has limited its utility. Another type of solvents used in organic and catalytic reactions is supercritical fluids. e.g., supercritical carbon dioxide [28]. Although they are described as green solvents, the critical conditions needed for their use is still a limitation.

Ionic liquids (ILs) are a new class of compounds that have received wide attention as green solvent alternatives. The terms room temperature ionic liquids (RTILs), molten salt, nonaqueous ionic liquid, liquid organic salt and fused salt have all been used to describe these salts [29]. ILs have been known for a long time, but their use as solvents has recently become significant. ILs such as [EtNH_3_][NO_3_] was first described in 1914 [30]. In the early 1970s, ILs were initially developed by electrochemists, for use as battery electrolytes [31]. Recently, ILs have found applications such as electrolytes for electrochemical devices and processes, homogeneous and heterogeneous catalysis, purification of gases, enzyme catalysis, biological reaction media and removal of metal ions [31]. As alternatives to volatile organic solvents (VOCs), ILs show nonvolatility, nonflammability, as well as high thermal and chemical stability [32]. Despite the aforementioned properties which make them environmentally friendly, an assessment of their impact on the environment and health is required to establish the safe use of some of them. For example, some recent reports have suggested that the ecotoxicity of alkyl methyl imidazolium cations used in biocatalysis increases with alkyl chain length in cation [33].

ILs can be prepared with different cation and anion combinations. Consequently the physical and chemical properties of ILs can be tuned according to the cation and anion used in the preparation. The properties of ILs are determined by mutual fit of cation and anion, size, geometry and charge distribution. Properties such as acidity, basicity, water miscibility and immiscibilty, hydrophilic and hydrophobic properties all result from the composite properties of cations and anions.

**Figure 4:**
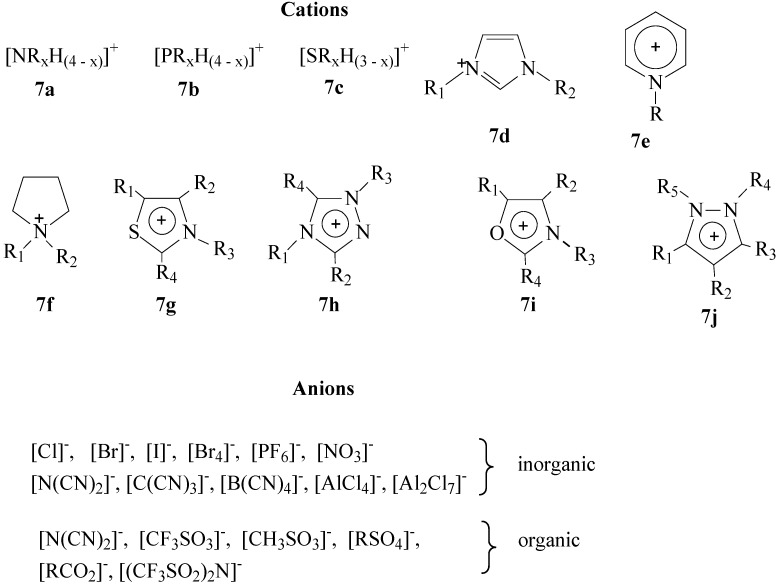
Examples of cations and anions described in ionic liquids.

The cation of the IL is generally a bulk organic structure with low symmetry. The most popularly used cations are ammonium (**7a**) [34,35,36], phosphonium (**7b**) [37], sulphonium (**7c**) [38], imidazolium (**7d**) [39,40,41,42,43], pyridinium **7e**) [44,45,46], pyrrolidinium (**7f**) [47], thiazolium (**7g**) [48], triazolium (**7h**) [49], oxazolium (**7i**) [50] and pyrazolium (**7j**) [51] cations (Figure 4). The most commonly used ILs are the salts based on the *N*,*N′*-dialkylimidazolium cation (**7d**). The melting point of IL decreases with the increase in size and asymmetry of the cation. Also an increase in the branching on the alkyl chain increases the melting point.

The anions present in the IL determine the hydrophobicity, viscocity, density and solvation of the IL system [52]. Different classifications have been used to describe IL anions, for example, as inorganic and organic anions as shown in Figure 4, or as fluorous anions such as PF_6_^−^, BF_4_^−^, CF_3_SO_3_^−^, (CF_3_SO_3_)_2_N^−^ and non-fluorous anions such as Al_2_Cl_7_^−^, Al_3_Cl_10_^−^, Au_2_Cl_7_^−^, Fe_2_Cl_7_^−^. The most widely used ILs are the ones with PF_6_^−^ and BF_4_^−^ anions. However, these two anions decompose when heated in presence of the water and produce HF. Anions such as CF_3_SO_3_^−^ and (CF_3_SO_3_)_2_N^−^ give thermally stable salts [53,54]. In these anions, the fluorine of the anion is bonded to carbon atom resulting in the C-F bond which is inert to hydrolysis.

### Properties of RTILs

RTILs are salts that are liquid at room temperature. They are also referred to as “designer solvents” since their solvent properties can be tuned for a specific application by varying the anion cation combinations. They have a unique array of physico-chemical properties, which make them superior, compared to the conventional organic solvents [55,56,57,58,59]. The main factors that influence the physical properties of RTILs are hydrogen bonding, charge distribution on the anions, polarity and dispersive interactions. Some of their basic properties are listed below [60]:

- Ability to dissolve many organic, inorganic and organometallic compounds.
- Immiscibility with many organic solvents.
- High polarity.
- Loosely coordinating bulky ions.
- Generally very low vapor pressures and low volatility.
- Thermal stability up to 300 °C approximately.
- High thermal conductivity and a large electrochemical window.
- Most have a liquid window of up to 200 °C which enables wide kinetic control.
- Nonaqueous polar alternatives for phase transfer processes.

## 5. Applications of RTILs in Metathesis of Oleochemical Feedstocks

### 5.1. Self-Metathesis

**Figure 5:**
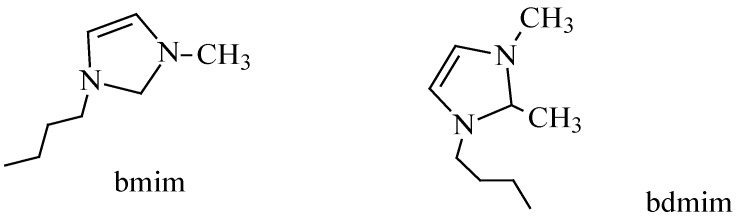
1-Butyl-3-methylimidazolium ion (bmim) and 1-butyl-2,3-dimethylimidazolium ion (bdmim).

**Scheme 4:**
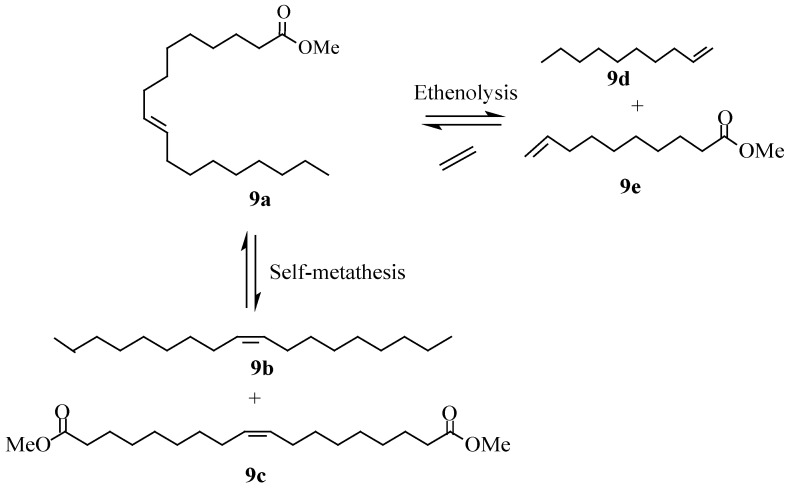
Methyl oleate metathesis.

**Scheme 5:**
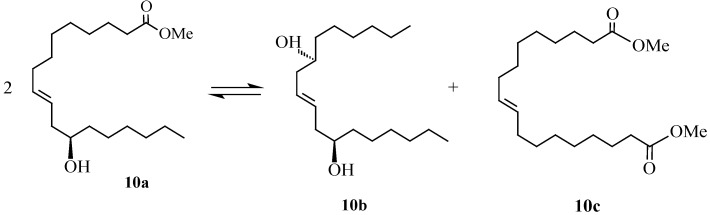
Self-metathesis of methyl ricinoleate.

The role of ionic liquids for the self-metathesis of seed oil derived olefins was investigated in our lab [41,61] using methyl oleate and methyl ricinoleate as subtrate esters. Self-metathesis of these oleochemical feedstocks with Grubbs and Hoveyda-Grubbs catalysts was carried out using 1,1-dialkyl and 1,2,3-trialkyl imidazolium type ionic liquids [bmim][X] where X = BF_4_^−^, PF_6_^−^ and NTf_2_^−^ and [bdmim][X] where X = BF_4_^−^ and PF_6_^−^. The structures for bmim and bdmim are shown in Figure 5. Scheme 4 and Scheme 5 show the metathesis products of methyl oleate (**9a**), namely, 9-octadecene (**9b**) and dimethyl 9-octadecenedioate (**9c**) and the metathesis products of methyl ricinoleate (**10a**), which are 9-octadecene-7,12-diol (**10b**) and dimethyl 9-octadecenedioate (**10c**).

Activity studies have shown that the anionic moiety does influence catalytic activity besides its influence on physical properties such as viscosity, density, hydrophobicity and solvation of ILs. The catalytic activity of Grubbs first generation catalyst (**5a**) for example, changes with varying anionic moiety in the following order: [bdmim][BF_4_] > [bdmim][PF_6_] > [bmim][BF_4_] > [bmim][NTf_2_] > [bmim][PF_6_]. Studies further record optimum activities and selectivies at moderate reaction temperatures not exceeding 60 °C.

Grubbs second generation catalyst exhibits higher methyl oleate conversions with improved selectivity in [bmim][X] ionic liquids over conventional organic solvents (Table 1) [41]. The higher activity of Grubbs second generation catalyst is attributed to the stabilization of the metallacycle intermediate by the more bulky and basic *N*-heterocyclic carbene ligand. Methyl oleate also exhibit high conversions (Table 2) when the metathesis reaction is carried out using Hoveyda-Grubbs second generation catalyst (**5d**). In particular higher substrate conversions were obtained in [bdmim][BF_4_] compared to [bmim][BF_4_] with excellent selectivity (>99%) observed at moderate reaction temperatures [61]. Methyl ricinoleate (methyl (9*Z*,12*R*)-12-hydroxyoctadec-9-enoate), whose fatty acid moiety constitutes about 90% of fatty acids in castor oil, also undergoes self-metathesis in ILs using Grubbs-type catalysts. Ricinoleate ester coversions in [bmim][PF_6_], [bmim][BF_4_] and [bmim][NTf_2_] are shown in Table 3.

Consorti *et al*. [62] have reported the transformation of methyl (ethyl) linoleate and soya bean oil into their conjugated isomers by RuHCl(CO)(PPh_3_)_3_ or RhCl(PPh_3_)_3_ in ILs. The ILs used in the study were [bmim][PF_6_], [bmim][NTf_2_], [bmPy][PF_6_] and [bmPy][NTf_2_]. A conversion of 80% was obtained when methyl linoleate was catalyzed using RuHCl(CO)(PPh_3_)_3_ in [bmim][NTf_2_] at 80 °C. A complete conversion of methyl or ethyl linoleate was observed with RhCl(PPh_3_)_3_/SnCl_2_ in [bmim][NTf_2_].

**Table 1:** Activity and selectivity of Grubbs catalysts **5a** and **5b** on methyl oleate in ILs *vs.* selected organic solvents.

Solvent/RTIL	Temperature/°C	Conversion/%	Selectivity/%	Substrate/Ru
Catalyst **5a**				
DCM	20	49	100	10^2^
	100	59	71	
DCE	20	51	100	10^2^
[bmim][PF_6_]	20	50	100	10^4^
	60	59	99	
[bmim][BF_4_]	20	54	100	10^4^
	60	61	95	
[bmim][NTf_2_]	20	51	100	10^4^
	60	61	99	
Catalyst **5b**				
DCM	20	52	100	10^2^
	120	81	27	
DCE	20	51	100	10^2^
[bmim][PF_6_]	20	53	100	10^4^
	60	75	81	
[bmim][BF_4_]	20	59	100	10^4^
	60	78	87	
[bmim][NTf_2_]	20	61	99	10^4^
	60	73	72	

Abbreviations: DCM (dichloromethane), DCE (Dichloroethane); Reference [41].

**Table 2:** Activity and selectivity of Hoveyda-Grubbs catalysts **5c** and **5d** on methyl oleate in ILs.

RTIL	Temperature/°C	Conversion/%	Selectivity/%	Substrate/Ru
Catalyst **5c**				
[bmim][PF_6_]	40	57	99	10^2^
	60	60	97	
[bmim][BF_4_]	40	58	99	10^2^
	60	60	97	
Catalyst **5d**				
[bdmim][PF_6_]	40	81	98	10^2^
	60	90	95	
[bdmim][BF_4_]	40	85	99	10^2^
	60	92	95	

Reference [61].

**Table 3:** Activity and selectivity of Hoveyda-Grubbs catalysts **5c** and **5d** on methyl ricinoleate in ILs.

RTIL	Temperature/°C	Conversion/%	Selectivity/%	Substrate/Ru
Catalyst **5a**				
[bmim][PF_6_]	60	45	99	10^2^
[bmim][BF_4_]	60	41	99	
[bmim][NTf_2_]	60	38	99	
Catalyst **5b**				
[bdmim][PF_6_]	60	58	99	10^2^
[bmim][BF_4_]	60	56	99	
[bdmim][NTf_2_]	60	55	99	10^2^

Reference [61].

### 5.2. Ethenolysis

**Figure 6:**
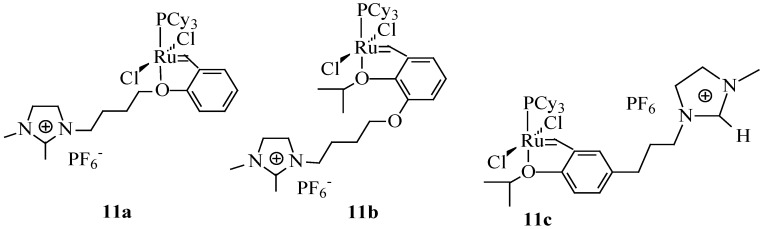
Hoveyda-type catalysts bearing an ionic tag [63].

Cross-metathesis of oil derived olefins with ethylene provides an interesting route for the synthesis of short-chain intermediates with potential application in polymer, lubricant and surfactant industries [63]. Ethenolysis of methyl oleate (**9a**) gives rise to 1-decene (**9d**) and methyl 9-decenoate (**9e**) as shown in Scheme 4. 1-Decene has numerous industrial applications, for example, it is used as an intermediate in the production of epoxides, amines, oxo alcohols, synthetic lubricants, synthetic fatty acids, and alkylated aromatics and as a monomer in co-polymers. Methyl 9-decenoate is an intermediate in the synthesis of many chemical products, for example, nylon-10 as well as lubricants and plasticizers [63]. Thurier *et al*. [64] reported the ethenolysis of methyl oleate in imidazolium-type ionic liquids using Grubbs and Hoveyda type catalysts. When three imidazolium based ILs ([bmim][OTf], [bmim][NTf_2_] and [bdmim][NTf_2_] were evaluated at room temperature in the presence of catalyst **5a**, the highest conversion (83%) was obtained in [bdmim][NTf_2_]. Hoveyda-type catalysts bearing an ionic tag (Figure 6) were also evaluated in the presence of [bdmim][NTf_2_]. Catalyst **11a** gave a better catalytic activity (89%) compared to **11b** although its recyclability was poor.

### 5.3. Cross-Metathesis in Biphasic Systems

**Figure 7:**
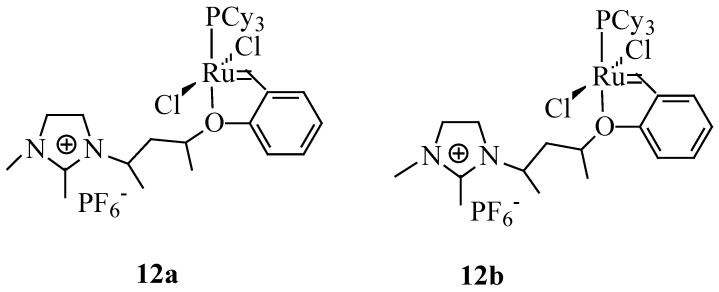
Ru-imidazolium-tagged carbene complexes [65].

Ethenolysis of methyl oleate using Ru-imidazolium-tagged carbene complexes (**12a**,**b**) (Figure 7) in biphasic systems with ILs have been reported by Aydos *et al*. [65]. A substrate conversion of 62% accompanied with the best selectivity of 46% was reported for the ionic tagged catalyst **12b** in [bmim][PF_6_] and toluene as the co-solvent. The addition of organic solvents to IL reduces the viscosity of the mixture and influences the transport properties and gas bubbling capability of ILs. To solve the problems associated with ionic phase mass transfer, the reaction was performed using Silica Aerosil 200 as the solid support. This modification resulted in a significant increase in catalytic activity. However, the solid support used did not influence the selectivity for ethenolysis. The supported ionic liquid phase (SILP) catalyst prepared with the IL 1-isopentyl-3-methylimidazole hexafluorophosphate [ipmim][PF_6_] and silica showed a turnover number almost twice that of biphasic systems).

### 5.4. Ring Closing Metathesis

**Scheme 6:**
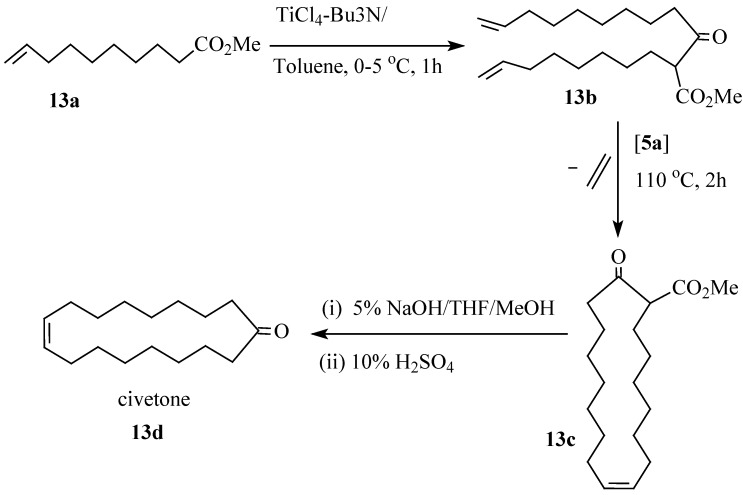
Synthesis of civetone via Ti-Claisen condensation and RCM.

**Scheme 7:**
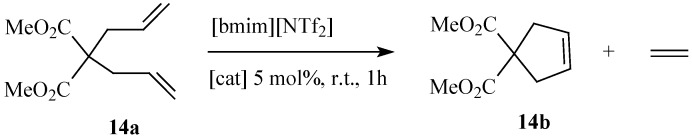
RCM of dimethyl diallylmalonate in [bmim][NTf2].

Ring closing metathesis has been successfully employed in the synthesis of macrocyclic compounds from olefinic fatty compounds in conventional organic solvents. For example civetone, an important ingredient in musk perfume can be synthesized by RCM of oleon (9,26-pentatriacontadien-18-one) which is first obtained by Claisen condensation of methyl oleate [66]. Alternatively, civetone could be synthesized in good yields via Ti-Claisen condensation of methyl 9-decenoate (Scheme 6) followed by RCM of β-ketoester **13b** using Grubbs catalyst **5a** to form a 17 membered β-ketoester **13c** which gets subjected to NaOH hydrolysis and acidic decarboxylation to form civetone **13d** [67]. However, RCM of diallyl esters in RTILs have also been reported using catalysts **11a** and **11b** [68]. As a model substrate, dimethyl diallylmalonate was subjected to RCM in [bmim][NTf_2_] under mild reaction conditions (Scheme 7). Catalyst **11b** displayed a rather better activity than catalyst **11a** although the latter maintained an almost constant activity upon recycling. Conversely, catalyst **11b** showed a decrease in activity with the number of catalytic cycles. 

**Scheme 8:**
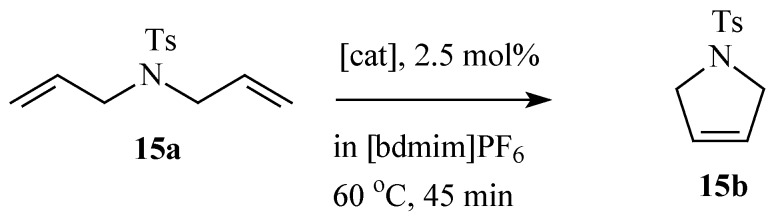
RCM of *N*,*N*-diallyltosylamide in [bdmim][PF6].

Ruthenium imidazolium tagged catalyst **11b** also shows excellent activity for the RCM of *N*,*N*-diallyltosylamide (Scheme 8) in the presence of [bdmim][PF_6_] resulting in the formation of cyclic olefin **15b** [68]. However, due to the difficulty in reforming the initial complex, catalytic activity drops drastically with the number of catalytic cycles while the activity of **11a** remains almost constant up to the second cycle and drops gently with further recycling. Audic *et al*. [69] have also reported the RCM of *N*,*N*-diallyltosylamide in [bmim][PF_6_] using catalyst **11c**. The above developments present interesting possibilities for applications of RTILs in RCM of oleochemicals.

### 5.5. Metathesis Using Supported Ionic Liquid Phase

**Scheme 9:**
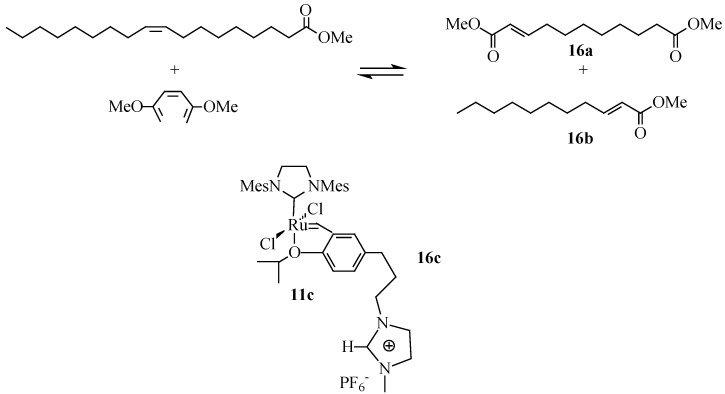
Cross-metathesis of methyl oleate with dimethyl maleate using **16c**.

Supported ionic liquid phase (SILP) catalysts are a combination of metal catalyst, ionic liquid and a porous support. Dugue *et al*. [70] reported a continuous flow homogeneous olefin metathesis using a SILP catalyst. A SILP catalyst (**16c**) immobilized on a thin film of [bmim][NTf_2_] and supported within the pores of silica, was used with compressed CO_2_ as the flowing medium. This system was applied for the self-metathesis of methyl oleate leading to a conversion of up to 64% and a turn over number of >10,000 was obtained after 9 h. The same SILP system was used for the cross-metathesis of methyl oleate with dimethyl maleate (Scheme 9) [70]. The reaction yields an unsaturated α,ω-diester (**16a**) and an α,β-unsaturated terminal ester (**16b**) which when carbonylated leads to the formation dimethyl 1,12-dodecandioate. These are all important feedstocks for applications in polymer synthesis [71,72,73].

### 5.6. Limitations with RTILs

The main limitations faced by RTILs are high cost and lack of physical property and toxicity data. They are comparatively more expensive than organic solvents and their cost depend on the composition of cations and anions and the scale of production [74]. The expensive cleaning methods due to their non-volatile nature and low melting points is another contributory factor to their high cost. Also, the desired RTIL quality (halide, water and amine content) may affect the price.

The non-availability of toxicity and biodegradability data is another challenge for the industrial scale application of RTILs [74,75]. Incomplete physico-chemical data also pose problems for the future use of RTILs. The data that is available currently is mainly on physical properties such as viscosity, density and phase transitions. Until recently, very few microscopic physical properties are available which is needed for the synthesis of new RTILs.

The recycling processes of RTILs available currently involves washing with water or volatile organic compounds, and the latter pose a threat to the environment. However, the application of supercritical extraction technologies and membrane separation processes is a promising intervention in the recycling RTILs. Still, some solid impurities are found to be adsorbed on the RTILs, which need to be eliminated using new technologies. The high viscosities further pose problems for its large scale application [76,77]. The viscosities of RTILs are higher than those of conventional organic solvents and similar to that of oils. The high viscosities may lead to a decrease in the rate of many organic reactions. Increasing the temperature and changing the anion-cation combinations can lower the viscosity of RTILs.

## 6. Future Directions

The introduction of room temperature ionic liquids to the transition metal catalysed reactions instead of conventional organic solvents has made the process more compatible to the environment and is found to be economical due to their unique physical and chemical properties such as non-volatility, excellent thermal stability, the ease of recovery and good ability to dissolve many organometallic compounds. In most of the publications reported, the use of ionic liquids in metathesis has led to significant enhancement in the reactivity, yield and reaction rate. Another advantage of using ionic liquids is the ease of recovering and reusing the catalyst and the ionic liquids without much drop in activity [78]. The ability to manipulate structure and function of ILs affords possibilities for designing a new generation of solvents with improved greener qualities and a wider industrial application [79].

Recent developments in olefin metathesis have promised a great future for the “green” metathesis in ionic liquids both in scientific research and in industrial and pharmaceutical applications. In spite of all these developments, many critical areas in catalytic olefin metathesis remain to be modified. The synthesis of more robust and active catalysts that can be easily prepared requires attention. The preparation of catalysts that can control olefin stereochemistry remains a challenge. Although many of the metathesis reactions yield the desired products, the high catalyst loadings required for an efficient and cost effective process is also under question.

## 7. Conclusions

Olefin metathesis is a ground-breaking advance that has cleared the route to many difficult chemical syntheses and is likely to continue to do so. The major disadvantages of the Ru catalysts are their poor recyclability and the difficulty of removing the Ru waste from the products. However, by the introduction of RTILs as solvents, the task of separating Ru catalysts from the products could be overcome. High conversions can therefore be achieved, and the catalyst can be reused and recycled. To overcome the problem of leaching of the catalyst into the organic phase, charged catalyst systems have been successfully employed. These charged catalysts improve the immobilization of the catalyst by increasing catalyst affinity to the ionic liquid. Furthermore, RTILs provide less harzadous and environmentally friendly reaction media. Nonetheless, a few limitations related to their high cost, availability of toxicity data and high viscosities should be taken into consideration before their large scale application.

## References

[1] Banks R.L., Bailey G.C. (1964). Olefin disproportionation: A new catalytic process. Ind. Eng. Chem. Prod. Res. Dev..

[2] Calderon N., Chen H.Y., Scott K.W. (1967). Olefin metathesis—A novel reaction for skeletal transformations of unsaturated hydrocarbons. Tetrahedron Lett..

[3] Mol J.C. (2002). Applications of olefin metathesis in oleochemistry: An example of green chemistry. Green Chem..

[4] Mol J.C. (2004). Catalytic metathesis of unsaturated fatty acid esters and oils: Catalytic conversion of renewables. Top. Catal..

[5] Plugge M.F.C., Mol J.C. (1991). A new synthesis of civetone. Synlett.

[6] Ivin K.J. (1998). Some recent applications of the olefin metathesis in organic synthesis: A review. J. Mol. Catal. A Chem..

[7] Copéret C. (2011). Stereoselectivity of supported alkene metathesis catalysts: A goal and a tool to characterize active sites. Beilstein J. Org. Chem..

[8] Loupy A., Tchoubar B., Astruc D. (1992). Salt effects resulting from exchange between two ion-pairs and their critical role on reaction pathways. Chem. Rev..

[9] Hérisson J.-L., Chauvin Y. (1971). Catalyse de transformation des olefins par les complexes du tungsténe II. Télomérisation des olefins cycliques en presence dʹoléfines acycligues. Makromol. Chem..

[10] De Espinosa L.M., Meier M.A.R. (2012). Olefin metathesis of renewable platform chemicals. Top. Organomet. Chem..

[11] Fischmeister C., Bruneau C. (2013). RTILS in catalytic olefin metathesis reactions. Top. Organomet. Chem..

[12] Miao S., Wang P., Su Z., Zhang S. (2014). Vegetable-oil-based polymers as future polymeric biomaterials. Acta Biomater..

[13] Lligadas G., Ronda J.C., Galià M., Cádiz V. (2013). Renewable polymeric materials from vegetable oils: A perspective. Mater. Today.

[14] Meier M.A.R. (2009). Metathesis with oleochemicals: New approaches for the utilization of plant oils as renewable resources in polymer science. Macromol. Chem. Phys..

[15] Hoveyda A.H., Zhugralin A.R. (2007). The remarkable metal-catalysed olefin metathesis reaction. Nature.

[16] Sanford M.S., Ulman M., Grubbs R.H. (2001). New insights into the mechanism of Ruthenium-catalyzed olefin metathesis reactions. J. Am. Chem. Soc..

[17] Sanford M.S., Love J.A., Grubbs R.H. (2001). Mechanism and activity of ruthenium olefin metathesis catalysts. J. Am. Chem. Soc..

[18] Adlhart C., Chen P. (2004). Mechanism and activity of ruthenium olefin metathesis catalysts: The role of ligands and substrates from a theoretical perspective. J. Am. Chem. Soc..

[19] Scott S.L. (2007). Catalytic transformation of seed oil derivatives via olefin metathesis. HELIA.

[20] Weskamp T., Schattenmann W.C., Spiegler M., Hermann W.A. (1998). A novel class of ruthenium catalysts for olefin metathesis. Angew. Chem. Int. Ed..

[21] Hoveyda A.H., Gillingham D.G., van Veldhuizen J.J., Kataoka O., Garber S.B., Kingsbury J.B., Harrity J.P.A. (2004). Ru complexes bearing bidentate carbenes: From innocent curiosity to uniquely effective catalysts for olefin metathesis. Org. Biomol. Chem..

[22] Śledź P., Mauduit M., Grela K. (2008). Olefin metathesis in ionic liquids. Chem. Soc. Rev..

[23] Cornils B., Herrmann W.A. (1998). Aqueous-Phase Organometallic Catalysis—Concept and Applications.

[24] Joo F., Beck M.T. (1975). Formation and catalytic properties of water-soluble phosphine complexes. React. Kinet. Catal. Lett..

[25] Kuntz E. (1987). Homogeneous catalysis in water. Chemtech.

[26] Horvath I.T., Rabai J. (1994). Facile catalyst separation without water: Flourous biphase hydroformylation of olefins. Science.

[27] Horvath I.T., Cornils B., Herrmann W.A. (1998). Flourous phases. Aqueous-phase Organometallic Catalysis-Concepts and Applications.

[28] Jessop P.G., Ikariya T., Noyori R. (1999). Homogeneous catalysis in supercritical fluids. Chem. Rev..

[29] Welton T. (1999). Room temperature ionic liquids: Solvents for synthesis and catalysis. Chem. Rev..

[30] Sugden S.H., Wilkins H. (1929). The parachor and chemical constitution. Part X11. Fused metals and salts. J. Chem. Soc..

[31] Gorman J. (2001). Faster, better, cleaner?: New liquids take aim at old-fashioned chemistry. Sci. News.

[32] Tavares A.P.M., Rodrígues O., Macedo E.A., Kadokawa J. (2013). New generations of ionic liquids applied to enzymatic biocatalysis. Ionic Liquids—New Aspects for the Future.

[33] Peric B., Martí E., Sierra J., Cruańas R., Garau M.A., Muńoz-Torrero D., Haro D., Vallés J. (2012). Green chemistry: Ecotoxicity and biodegradability of ionic liquids. Recent Advances in Pharmaceutical Sciences II.

[34] Bond D.R., Jackson G.E., Joao H.C., Hofmeyr M.N., Modro T.A., Nassimbeni L.R. (1989). Liquid clathrates. J. Chem. Soc. Chem. Commun..

[35] Hill M.G., Lamanna W.M., Mann K.R. (1991). Tetrabutylammonium tetrakis[3, 5-bis(trifluoromethyl)phenyl]borate as a non-coordinating electrolyte: Reversible 1e-oxidations of ruthenocene, osmocene, and Rh2(TM4)42+. Inorg. Chem..

[36] Sun J., Forsyth M., Mac Farlane D.R. (1998). Room temperature molten salts based on quartenary ammonium ion. J. Phys. Chem. B.

[37] Olivier-Bourbigou H., Magna L. (2002). Ionic liquids: Perspectives for organic and catalytic reactions. J. Mol. Catal. A Chem..

[38] Miyatake K., Yamamoto K., Endo K., Tsuchida E. (1998). Superacidified reaction of sulfides and esters for the direct synthesis of sulfonium derivatives. J. Org. Chem..

[39] Wilkes J.S., Levisky J.A., Wilson R.A., Hussey C.L. (1982). Dialkylimidazolium chloroaluminate melts: A new class of room temperature ionic liquids for electroscopy, spectroscopy and synthesis. Inorg. Chem..

[40] Fannin A.A., King L.A., Levisky J.A., Wilkes J.S. (1984). Properties of 1,3-dialkylimidazolium chloride-aluminium chloride ionic liquids. 1. Ion interactions by nuclear magnetic resonance spectroscopy. J. Phys. Chem..

[41] Thomas P.A., Marvey B.B. (2009). C18:1 methyl ester metathesis in [bmim][X] type ionic liquids. Int. J. Mol. Sci..

[42] Bonhˆote P., Dias A.P., Papageorgiou K., Kalyanasundaram K., Grätzel M. (1996). Hydrophobic, highly conductive ambient-temperature molten salts. Inorg. Chem..

[43] Hurley F.H., Weir T.P. (1951). Electrodeposition of metals from fused quarternary ammonium salts. J. Electrochem. Soc..

[44] Gale R.J., Osteryoung R.A. (1980). Infrared spectral investigations of room-temperature aluminium chloride-1-butylpyridinium chloride melts. Inorg. Chem..

[45] Tait S., Osteryoung R.A. (1984). Infrared study of ambient-temperature chloroaluminates as a function of melt acidity. Inorg. Chem..

[46] Mac Farlane D.R., Meakin P., Sun J., Amini N., Forsyth M. (1999). Pyrrolidinium imides: A new family of molten salts and conductive plastic crystal phases. J. Phys. Chem. B..

[47] Davis J.H., Forrester K.J. (1999). Thiazolium-ion based organic ionic liquids. Tetrahedron Lett..

[48] Vestergaard B.B., Petrushina N.J.I., Hjuler H.A., Berg R.W., Begtrup M. (1993). Molten triazolium chloride systems as new aluminium battery electrolytes. J. Electrochem. Soc..

[49] Jorapur Y.R., Chi D.Y. (2006). Ionic Liquids: An Environmentally Friendly Media for Nucleophilic Substitution Reactions. Bull. Korean Chem. Soc..

[50] Hussey C.L., Mamantov G., Popov A.I. (1994). The electrochemistry of room temperature haloaluminate molten salts. Chemistry of Nonaqueous Solutions: Current Progress.

[51] Brennecke J.F., Maginn E.J. (2001). Ionic liquids: Innovative fluids for chemical processing. AIChE J..

[52] Garcia M.T., Gathergood N., Scammells P.J. (2005). Biodegradable ionic liquids Part II. Effect of the anion and toxicology. Green Chem..

[53] Tao G.-H., He L., Sun N., Kou Y. (2005). New generation ionic liquids: Cations derived from amino acids. Chem. Commun..

[54] Yang Q., Dionysiou D.D. (2004). Photolytic degradation of chlorinated phenols in room temperature ionic liquids. J. Photochem. Photobiol. A: Chem..

[55] Seddon K.R. (1996). Room-temperature ionic liquids-neoteric solvents for clean catalysis. Kinet. Catal..

[56] Lagrost C., Carrié D., Vaultier M., Hapiot P. (2003). Reactivities of some electrogenerated organic cation radicals in room-temperature ionic liquids: Toward an alternative to volatile organic solvents?. J. Phys. Chem. A.

[57] Shariati A., Peters C.J. (2005). High-pressure phase equilibria of systems with ionic liquids. J. Supercrit. Fluids.

[58] Shariati A., Gutkowski K., Peters C.J. (2005). Comparison of the phase behavior of some selected binary systems with ionic liquids. AIChE J..

[59] Zhao H., Xia S., Ma P. (2005). Use of ionic liquids as green solvents for extractions. J. Chem. Technol. Biotechnol..

[60] Keskin S., Kayrak-Talay D., Akman U., Hortaçsu Ö. (2007). A review of ionic liquids towards supercritical fluid applications. J. Supercrit. Fluids.

[61] Thomas P.A., Marvey B.B., Ebenso E.E. (2011). Metathesis of fatty acid ester derivatives in 1,1-dialkyl and 1,2,3-trialkyl imidazolium type ionic liquids. Int. J. Mol. Sci..

[62] Consorti C.S., Aydos G.L.P., Ebeling G., Dupont J. (2009). Multiphase catalytic isomerization of linoleic acid by transition metal complexes in ionic liquids. Appl. Catal. A.

[63] Khorasvi E., Snymanska-Buzar T. (2000). Ring opening metathesis polymerization and related chemistry. Nato Sci. Ser..

[64] Thurier C., Fischmeister C., Bruneau C., Olivier-Bourbigou H., Dixneuf P.H. (2008). Ethenolysis of methyl oleate in room-temperature ionic liquids. ChemSusChem..

[65] Aydos G.L.P., Leal B.C., Perez-Lopez O.W., Dupont J. (2014). Ionic-tagged catalytic systems applied to the ethenolysis of methyl oleate. Catal. Commun..

[66] Boelhouwer C., Mol J.C. (1984). Metathesis of fatty acid esters. J. Am. Oil Chem. Soc..

[67] Hamasaki R., Funakoshi S., Misaki T., Tanabe Y. (2000). A highly efficient synthesis of civetone. Tetrahedron.

[68] Thurier C., Fischmeister C., Bruneau C., Olivier-Bourbigou H., Dixneuf P.H. (2007). Ionic imdazolium containing ruthenium complexes and olefin metathesis in ionic liquids. J. Mol. Catal. A Chem..

[69] Audic N., Clavier H., Mauduit M., Guillemin J.-C. (2003). An ionic liquid-supported Ruthenium carbene complex: A robust and recyclable catalyst for ring-closing olefin metathesis in ionic liquids. J. Am. Chem. Soc..

[70] Duque R., Ochsner E., Clavier H., Caijo F., Nolan S.P., Mauduit M., Cole-Hamilton D.J. (2011). Continuous flow homogeneous alkene metathesis with built-in catalyst separation. Green Chem..

[71] Patel J., Mujcinovic S., Jackson W.R., Robinson A.J., Serelisand A.K., Such C. (2006). High conversion and productive catalyst turn overs in cross-metathesis reactions of natural oils with 2-butene. Green Chem..

[72] Jiḿenez-Rodriguez C., Eastham G.R., Cole-Hamilton D.J. (2005). Dicarboxylic acid esters from the carbonylation of unsaturated esters under mild conditions. Inorg. Chem. Commun..

[73] Patel J., Elaridi J., Jackson W.R., Robinson A.J., Serelisand A.K., Such C. (2005). Cross-metathesis of unsaturated natural oils with 2-butene. High conversion and productive catalyst turnovers. Chem. Commun..

[74] Kerton F. (2009). Alternative Solvents for Green Chemistry:RCS Green Chemistry Book Series.

[75] Sowmiah S., Srinivasadesikan V., Tseng M.-C., Chu Y.-H. (2005). On the chemical stabilities of Ionic liquids. Molecules.

[76] Andreussi O., Marzari N. (2012). Transport properties of RTILs from classical molecular dynamics. J. Chem. Phys..

[77] Ghandi K. (2014). A review of ionic liquids, their limits and applications. Green Sustain. Chem..

[78] Fishmeister C., Bruneau C., Dupont J., Kollar L. (2015). RTILs in catalytic olefin metathesis reactions. Ionic Liquids (ILs) in Organometallic Catalysis.

[79] Pagni R.M., Rogers R.D., Seddon R.K., Volkov S. (2003). Ionic liquids as alternatives to traditional organic and inorganic solvents. Green Industrial Applications of Ionic Liquids.

